# A case of fatal intoxication with the novel synthetic opioid *N*-pyrrolidino protonitazene

**DOI:** 10.1007/s00414-025-03618-8

**Published:** 2025-10-18

**Authors:** Sophia Wrbas, Tom R. Sundermann, Volker Auwärter, Laura M. Huppertz

**Affiliations:** 1 Forensic Toxicology, Institute of Forensic Medicine, Medical Center, Medical Faculty, University of Freiburg, University of Freiburg, Freiburg, Germany; 2 Forensic Toxicology, Institute for Legal Medicine and Traffic Medicine, Heidelberg University Hospital, University of Heidelberg, Heidelberg, Germany

**Keywords:** *N*-Pyrrolidine protonitazene, Protonitazepyne, Novel psychoactive substances (NPS), Nitazene, Standard addition method, LC-MS/MS

## Abstract

**Purpose:**

The presence of 2-benzylbenzimidazole-derived opioids (nitazenes) in the drug market is steadily increasing. These often highly potent synthetic opioids can cause severe, potentially fatal effects. This case report presents a fatal intoxication following the vaping of the novel synthetic opioid *N*-pyrrolidino protonitazene (protonitazepyne), detected in serum obtained 10 h postmortem and in multiple specimens collected at autopsy three days after death.

**Methods:**

Concentrations of *N*-pyrrolidino protonitazene in postmortem serum, heart blood, femoral blood, liver, bile, and stomach contents were determined using liquid chromatography–tandem mass spectrometry (LC–MS/MS) with a standard addition approach. Urine and serum were additionally analyzed via LC–MS/MS using external matrix calibration.

**Results and discussion:**

*N*-Pyrrolidino protonitazene was detected at 3.8 ng/mL in postmortem serum, 1.7 ng/mL in heart blood, approx. 0.52 ng/mL in femoral blood, 0.34 ng/g in liver, 32.3 ng/mL in bile, and 19.7 ng/mL in stomach contents. Urine contained 8.8 ng/mL. Additional findings in postmortem serum included pregabalin (7.2 µg/mL), sertraline (66 ng/mL), naloxone (10.2 ng/mL), mitragynine (11.5 ng/mL), and traces of quetiapine. These co-ingestants did likely not account for the fatal outcome. With an estimated in vitro potency approximately 25-fold greater than fentanyl, even low doses of N-pyrrolidino protonitazene can be lethal. Elevated heart-to-femoral blood ratios indicate postmortem redistribution, while declining levels over time may indicate compound instability.

**Conclusion:**

Due to its high potency, *N*-pyrrolidino protonitazene is considered the primary cause of death. Accordingly, a toxicological significance score of 3 is assigned, underscoring the potential public health risk posed by this novel synthetic opioid.

## Introduction

2-Benzylbenzimidazole opioids, also known as nitazenes, are part of the rapidly expanding class of Novel Synthetic Opioids (NSOs), which represent one of the largest and most dynamic groups within New Psychoactive Substances (NPS), with new compounds continuously emerging. NSOs first emerged on the drug market in 2009 and have continued to proliferate ever since. Between 2009 and 2022, 74 different NSOs were detected in the European Union [1].

This subclass of 2-benzylbenzimidazoles was initially developed and patented by the Swiss pharmaceutical company CIBA in the 1950 s as potent analgesics. However, they were not pursued for medical use due to their potentially fatal side effects, such as sedation or respiratory depression [2–4].

Nitazenes resurfaced in illicit drug markets in 2019, following the international scheduling of fentanyl in the United States and China. Since then, their prevalence has steadily increased, as documented by the European Union Drugs Agency (EUDA), formerly known as European Monitoring Centre for Drugs and Drug Addiction (EMCDDA) [1, 2, 5].

Although structurally unrelated to fentanyl and morphine, nitazenes exhibit similar toxicity and adverse effects, ranging from fatigue, nausea, and vomiting to respiratory depression, coma, and fatal overdose. Due to their structural novelty, newly synthesized nitazenes often evade existing drug legislation, thereby serving as “legal” alternatives to internationally controlled substances [2, 5–7].

Nitazenes are available in various forms - including powders, tablets, and liquids -, and are often used simultaneously with other substances such as fentanyl, heroin, or benzodiazepines [8].

**Fig. 1:**
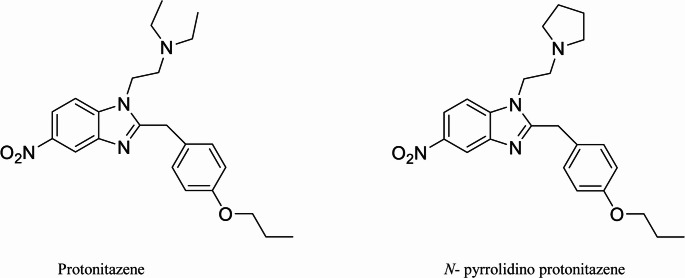
Protonitazene *N*- pyrrolidino protonitazene

Examples for 2-benzylbenzimidazole opioids include etonitazene, isotonitazene and protonitazene (Fig. 1). These compounds have been reported to be up to 1000 times more potent than morphine, with etonitazene currently recognized as the most potent nitazene identified. The analgesic effects of this class are mediated through their action as µ-opioid receptor (MOR) agonists. Research suggests that the nitro group at the 5-position of the benzimidazole ring is a key contributor to their high potency [2, 3, 7, 9].

This case report focuses on the 2-benzylbenzimidazole derivative *N*-pyrrolidino protonitazene (Fig. 1), also known as protonitazepyne, which emerged on the European drug market in mid-2023. In this instance, the consumed nitazene was misidentified and sold under the name “protonitazene.” In vitro studies demonstrated that *N*-pyrrolidino protonitazene has approximately 25 times the potency of fentanyl, while protonitazene is about 3.5 times more potent than fentanyl. Consequently, *N*-pyrrolidino protonitazene seems to exhibit an in vitro potency roughly seven times greater than protonitazene. Due to its high potency, *N*-pyrrolidino protonitazene has been involved in a number of fatal cases. Reported postmortem blood concentrations ranged from 0.1 to 55 ng/mL, with a mean concentration of 6.9 ng/mL [10–14].

In this report, we present and analyze a fatal intoxication case that occurred in Germany in July 2023. We discuss the concentrations of *N*-pyrrolidino protonitazene found across multiple biological matrices, its toxicological effects, and relevant postmortem findings.

## Case history

In July 2023, a 23-year-old man was found lifeless in a room at a social accommodation facility. According to police records, he had consumed drugs with friends the previous evening, including “protonitazene,” which they used in an e-cigarette. Later that night, the man lay down on a bed, where his friends initially observed him snoring but later noticed his breathing becoming increasingly shallow. Despite emergency resuscitation efforts, he was finally pronounced dead. The attending physician classified the death as non-natural. Witnesses reported that the man had a history of drug dependency but had successfully completed rehabilitation. However, he had recently begun experimenting with substances again. Key autopsy findings included cerebral edema, hemorrhagic pulmonary edema, an abundance of liquid blood, an overfilled bladder, and generalized organ congestion - all indicative of fatal drug intoxication. A serum and a NaF-preserved plasma sample were collected shortly after death. During the autopsy conducted three days postmortem, biological samples including femoral blood, heart blood, and urine were collected for subsequent analysis. Furthermore, two urine samples of the deceased, originating from an abstinence monitoring program, performed from March to May 2023, were later screened for *N*-pyrrolidino protonitazene and other opioids. A plasma sample taken from a co-consumer of the deceased was also analyzed.

## Materials and methods

### Reference materials and chemicals

The reference standard of *N*-pyrrolidino protonitazene and the internal standard fentanyl-D_5_ were purchased from Cayman Chemical (Ann Arbor, MI, USA) and LGC Standards GmbH (Wesel, Germany). Methanol (Chromasolv™ LC-MS grade) was bought from Honeywell (Seelze, Germany). Acetonitrile (LC-MS grade) was from VWR Chemicals (Darmstadt, Germany). Ammonium formate 10 M in H_2_O was purchased from Sigma-Aldrich (Steinheim, Germany). Formic acid (≥ 98%, p.a., ACS) was from Carl Roth (Karlsruhe, Germany). Deionized water was obtained by using a Medica^®^ Pro deionizer from ELGA (Celle, Germany). The *N*-pyrrolidino protonitazene research chemical with an 80% purity level, as determined by NMR, was provided by Netzwerk ADEBAR (Kiel, Germany).

### LC-MS/MS method

Chromatography was performed as described by Berardinelli et al. [2] Briefly, the analysis was performed on a Shimadzu Nexera X2 LC-30AD (Duisburg, Germany) coupled to a Sciex QTRAP 5500 triple quadrupole linear iron trap instrument (Darmstadt, Germany). Separation was achieved using a Phenomenex Kinetex^®^ F5 column (2.6 μm, 100 × 2.1 mm) with an additional F5 pre-column (2.1 mm) with a flow rate of 0.5 mL/min. An adapted MS method containing the following MRMs was used for quantification of *N*-pyrrolidino protonitazene: 409.3/409.3, 409.3/149.1 and 409.3/98.0. Fentanyl-D_5_ was used as internal standard (MRM transition: 342.0/188.2).

### Preparation of urine and serum samples

Blood and urine samples were prepared as follows. For serum and plasma preparation, 100 µL serum or plasma was combined with 100 µL of ammonium formate solution (10 M), 10 µL internal standard (fentanyl-D_5_, 100 ng/mL), and 1 mL ice-cold acetonitrile. The samples were shaken for 5 min and then centrifuged for 10 min at 2896 g. The supernatant was transferred to a vial and evaporated to dryness under a gentle nitrogen stream at 40 °C. The residue was reconstituted in 100 µL of a mobile phase mixture consisting of mobile phase A (1% acetonitrile, 0.1% formic acid and 2 mM ammonium formate in water) and mobile phase B (0.1% formic acid and 2 mM ammonium formate in acetonitrile) in a 95:5 v/v ratio. For analysis, 10 µl were injected into the liquid chromatography-tandem mass spectrometry (LC-MS/MS) system. Urine samples were prepared using the same procedure, with the addition of 100 µL phosphate buffer (pH6) and adjusting reagent volumes to 200 µL ammonium formate (10 M) and 1.5 mL ice-cold acetonitrile.

Urine and serum samples were quantified using a standard calibration curve with the following concentrations: 1 ng/mL, 5 ng/mL, 10 ng/mL, 15 ng/mL and 20 ng/mL. All of the calibrants were prepared in a serum or urine matrix. Bovine serum was used for serum calibration, and urine donated by volunteers and checked for the absence of drugs and drugs of abuse was used for urine calibration. A serum calibration was used to quantify the plasma samples. Additionally, the plasma obtained from the deceased was quantified before a certified reference standard became available. Therefore, the concentration value was corrected for the 80% purity of the research chemical used to prepare the reference substance solution, which resulted in increased measurement uncertainty. Due to the very limited amount of sample material a re-analysis was not possible.

### Standard addition method

The standard addition method was employed to determine the concentrations of *N*-pyrrolidino protonitazene in various body fluids and tissues. This method was applied following the protocols described by Hasegawa et al. and Giorgetti et al. [15, 16]. Before quantification, semiquantitative analyses were conducted to estimate the concentration of *N*-pyrrolidino protonitazene in serum and urine samples.

The standard addition method was applied to postmortem blood, bile, stomach contents, and liver samples. For blood, bile, and stomach content samples, 100 µL aliquots were used. For liver samples, three aliquots of approximately 500 mg were weighed and homogenized with 500 µL of phosphate buffer (pH 6) using a BeadBug™ microtube homogenizer (Benchmark Scientific, UK). The homogenized samples were pooled and diluted with an additional 1.5 mL of phosphate buffer. The mixture was centrifuged, and the supernatant collected. Aliquots of 100 µL from the supernatant were then used for the standard addition method. The samples were processed following the previously described protocol.

For each matrix a six-point calibration curve was established, including a zero sample containing only matrix and internal standard. The calibration curve consisted of the following spiked concentrations: 1 ng/mL, 5 ng/mL, 7.5 ng/mL, 10 ng/mL, and 15 ng/mL. To quantify the *N*-pyrrolidino protonitazene in bile, an additional calibration curve was used with the following concentrations: 20 ng/mL, 30 ng/mL, 50 ng/mL, 75 ng/mL, and 100 ng/mL. For liver samples, the calibration curve included the following absolute concentrations per sample: 100 pg, 500 pg, 750 pg, 1,000 pg, and 1,500 pg. Each liver sample contained a total of 65 mg of tissue in 100 µL of solution.

## Results and discussion

### Postmortem examination

The autopsy of the deceased was performed three days postmortem, and macroscopically revealed no clearly identifiable cause of death. As a result, the manner and cause of death initially remained undetermined. The pathological findings were cerebral edema, characterized by swelling of the brain tissue, hemorrhagic pulmonary edema, indicating fluid accumulation in the lungs accompanied by blood congestion, a markedly distended (full) urinary bladder, and a noticeable congestion of the internal organs. While these findings are non-specific, their combination is frequently observed in fatal intoxications. Additionally, mild generalized arteriosclerosis, indicative of early-stage arterial hardening, was detected. Additionally, a mild left ventricular hypertrophy was noted, as well as suspected purulent secretion in the right palatine tonsil, which may indicate an underlying infection.

Since the autopsy did neither reveal any competing, significant internal organ pathology that could independently explain the cause of death nor any evidence of severe mechanical trauma inflicted by external force and considering the case history, a fatal intoxication was suggested by the forensic pathologists. Hence, the manner of death was ruled non-natural.

### Toxicological analyses

Toxicological screening of postmortem serum and NaF-plasma revealed *N*-pyrrolidino protonitazene, along with pregabalin, sertraline, quetiapine, naloxone, and mitragynine. The measured naloxone concentration can be attributed to admission during resuscitation by emergency services. According to reports, quetiapine was given to the deceased by a co-consumer. However, there was no information available regarding the antidepressant sertraline, the anticonvulsant pregabalin, or the use of mitragynine. Although pregabalin and sertraline concentrations are elevated, they do not reach toxic levels and remain within the range compatible with therapeutic use. While *N*-pyrrolidino protonitazene was detected in the postmortem urine sample, the urine samples from the abstinence monitoring program tested positive only for mitragynine. Neither *N*-pyrrolidino protonitazene, protonitazene, nor any other currently available 2-benzylbenzimidazole derivative opioids were detected. The plasma sample from a co-consumer was also tested and confirmed positive for *N*-pyrrolidino protonitazene. All serum and urine sample concentrations, quantified through external calibration, are listed in Table 1.

**Table 1:** Toxicological screening results

Matrix	Substance	Concentration
Postmortem serum	*N*-Pyrrolidino protonitazene	7.8 ng/mL
	Naloxone	10.2 ng/mL
	Mitragynine	11.5 ng/mL
	Pregabalin	7.2 µg/mL
	Sertraline	66 ng/mL
	Quetiapine	< 50 ng/mL
Postmortem urine	*N*-Pyrrolidino protonitazene	8.8 ng/mL
	Creatinine	200 mg/dl
NaF-Plasma	*N*-Pyrrolidino protonitazene	5.9 ng/mL*
NaF-plasma, co-consumer	*N*-Pyrrolidino protonitazene	1.2 ng/mL

* Concentration values were corrected for the purity of approximately 80% of the research chemical used to prepare the reference substance solution. Therefore, increased measurement uncertainty is possible, but the correct order of magnitude should be reflected.

To mitigate matrix effects commonly observed in postmortem samples, a standard addition method was performed for postmortem serum, heart blood, femoral blood, bile, stomach contents and liver samples. The concentrations of *N*-pyrrolidino protonitazene and corresponding quantitation equations, along with their correlation coefficients (r^2^) are presented in Table 2. Absolute drug concentration of *N*-pyrrolidino protonitazene in stomach contents (400 mL) was 7.88 µg.

**Table 2:** Concentrations of *N*-Pyrrolidino protonitazene quantified using SAM

Matrix	Concentration of *N*-pyrrolidino protonitazene	Equation	Correlation coefficient (*r*^2^)
Serum	3.83 ng/mL	y = 0.1631x + 0.6237	0.988
Heart blood	1.67 ng/mL	y = 0.0725x + 0.1215	0.986
Femoral blood	Approx. 0.52 ng/mL*	One point calibration	-
Bile	32.3 ng/mL	y = 0.6299x + 20.379	0.991
Stomach content	19.7 ng/mL (absolute 7.88 µg)	y = 0.4829x + 9.5262	0.985
Liver	0.34 ng/g	y = 0.0025x – 0,0568	0.994

* Arithmetic mean (*n* = 4; range: 0.39–0.65 ng/ml; relative standard deviation: 28%)

The variation in concentrations among postmortem serum, heart blood, and femoral blood samples analyzed using standard addition can be attributed to differences in sampling times and the associated postmortem interval. The serum sample was collected 10 h postmortem, whereas the other samples were collected during the autopsy 3 days later. During this period, postmortem redistribution and substance degradation may have occurred.

Postmortem redistribution refers to changes in drug concentrations after death, influenced by factors such as volume of distribution, lipophilicity, and pKa. The extent of redistribution increases over time, particularly in basic, lipophilic drugs, which are more prone to this process than water-soluble substances. A higher drug concentration in central (heart) blood compared to peripheral (femoral) blood, expressed as the central-to-peripheral concentration ratio (C/P ratio), suggests redistribution. A key mechanism of postmortem redistribution is the diffusion of drugs from tissue reservoirs with high concentrations to areas of lower concentrations following cellular membrane disruption during decomposition [17–20].

The C/P ratio of 2.3 for *N*-pyrrolidino protonitazene suggests postmortem redistribution. This is consistent with the chemical properties of nitazenes, which are significantly more lipophilic than morphine as demonstrated by calculated values for the n-octanol-water partition coefficients (log P) [14].

The elevated *N*-pyrrolidino protonitazene concentrations in bile and stomach contents align with documented reports of its consumption via vaping. High drug concentrations in bile are indicative of recent drug use and are commonly observed with this drug class. Additionally, the phenomenon of hepatobiliary disposition, also described for opiates, could account for the measured concentration of *N*-pyrrolidino protonitazene in bile [20, 21].

The discrepancy in *N*-pyrrolidino protonitazene concentrations between the postmortem serum samples analyzed through external calibration (7.8 ng/mL) versus the standard addition method (3.8 ng/mL) is attributable to matrix effects inherent in postmortem samples. These effects are minimized when using the standard addition method [11].

Given the high potency of *N*-pyrrolidino protonitazene relative to morphine, fentanyl, and protonitazene, the measured concentrations are considered highly toxic and potentially lethal. As *N*-pyrrolidino protonitazene was incorrectly labeled as “protonitazene”, and given its significantly greater in vitro potency in comparison to protonitazene, the mislabeling of the substance may have contributed to an unintended overdose with fatal outcome. Based on the toxicological findings, autopsy results, and existing literature on benzimidazole opioids, *N*-pyrrolidino protonitazene was determined to be the primary cause of death [5, 14]. However, pregabalin and mitragynine could be considered additional contributing factors to the lethal outcome. A toxicological significance score of 3 was assigned to *N*-pyrrolidino protonitazene, based on the criteria established by Elliott et al. [22]. The toxicological significance is a tool used to evaluate the risk associated with new psychoactive substances or other drugs, which assigns a score to a drug according to its toxicity and contribution to death. A score of 3 denotes that the substance is the main cause of death, whereas scores of 1 or 2 indicate either a contributory role or an alternative cause.

Due to their risk of causing severe intoxication - even when not combined with other central nervous system depressants - their accessibility at relatively low prices and the increased likelihood to cause unintentional overdoses because of their high potency, nitazenes demand intensified public health and forensic attention. The present case further reinforces the necessity of a thorough and multidisciplinary evaluation of all available data, especially in the absence of information on toxic doses or concentration levels, to accurately assess the potential contributory role of the respective substances in fatal cases.

## Conclusion

In this case of fatal intoxication, the novel synthetic opioid *N*-pyrrolidino protonitazene was identified as the primary cause of death and was assigned a toxicological significance score of 3. The relatively low concentrations detected in postmortem blood samples underscore the compound’s high potency and the substantial risk of overdose, even at small doses. The findings also suggest postmortem redistribution and substance degradation, which may complicate toxicological interpretation. This case illustrates the growing threat posed by emerging nitazene derivatives, which continue to appear on the illicit drug market. Given their potential for severe and fatal outcomes, along with the limited pharmacological and toxicological data available, heightened forensic awareness, analytical preparedness, and regulatory action are needed to mitigate the public health risks associated with these substances.
